# Analysis of Degradation of Electromigration Reliability of Au-Al and OPM Wire Bonded Contacts at 250 °C Using Resistance Monitoring Method

**DOI:** 10.3390/mi14030640

**Published:** 2023-03-12

**Authors:** Xueqin Li, Linchun Gao, Tao Ni, Jingnan Zhou, Xiaojing Li, Yifan Li, Lida Xu, Runjian Wang, Chuanbin Zeng, Bo Li, Jiajun Luo, Jing Li

**Affiliations:** 1 Institute of Microelectronics of the Chinese Academy of Sciences, Beijing 100029, China; 2 University of Chinese Academy of Sciences, Beijing 101408, China; 3 Key Laboratory of Science and Technology on Silicon Devices, Chinese Academy of Sciences, Beijing 100029, China

**Keywords:** Au-Al wire bonding, OPM structure, electromigration reliability, high temperature

## Abstract

The ongoing trend towards miniaturization and increased packaging density has exacerbated the reliability problem of Au-Al heterogeneous metal bonding structures in high-temperature environments, where extreme temperatures and high current pose a serious challenge. In order to address this issue, the present study aims to investigate the electromigration reliability of Au-Al bonding by comparing the conventional heterogeneous contacts with OPM structures, which are homogeneous contacts. A novel bonding layout was developed to precisely detect the resistance and obtain stage changes in electromigration. The experimental results demonstrated that the relative resistance shift of Au-Al bonding at 250 °C was 98.7%, while CrAu and NiPdAu OPM structures exhibited only 46.1% and 2.93% shifts, which suggests that the reliability of OPM structures was improved by a factor of 2.14 and 33.6, respectively. The degradation of Au-Al bonding was attributed to the large cracks observed at the bonding interface and lateral consumption of Al elements. In contrast, OPM structures only exhibited tiny voids and maintained a better bonding state overall, indicating that homogeneous metal contacts have better immunity to electromigration. Furthermore, this study also observed the polarity effect of electromigration and analyzed the impact of NiPdAu thickness on reliability. Overall, this research provides a novel approach and an insightful theoretical reference for addressing the bottleneck of high-temperature electromigration reliability in high-temperature sensor packaging.

## 1. Introduction

The use of high-temperature electronic sensor devices has become increasingly prevalent in various industries, including aerospace, deep space exploration, oil well drilling, nuclear power plants, and engines. Consequently, the operating temperature has emerged as a critical factor affecting electrical performance. Despite the growing demand for devices and chips capable of stable operation at extreme temperatures of approximately 250 °C, the development and manufacture of such devices has remained a challenge, limiting progress in industrial and academic spaces [1,2,3,4,5]. In the packaging of highly reliable sensors, wire bonding has been the primary electrical interconnection method for decades. Since the 1960s, wire bonding has been extensively utilized for electrical interconnections in 2D and 3D stacked packages and System in Package (SIP) implementations, solidifying its position as a leading method in the field of advanced packaging due to its established process, high flexibility, low cost, and adaptability [6].

The core predicament lies in the fact that the conventional Au-Al wire bonding relies on the contact between heterogeneous metals, which leads to weakened bond strength due to the diffusion of metal atoms into one another, eventually resulting in the creation of intermetallic compounds (IMCs) and Kirkendall voids [7,8]. This significantly reduces the operational lifespan of wire bonding, especially under conditions of high temperature. Furthermore, it has been demonstrated in previous studies [9,10,11] that electromigration accelerates the formation of IMCs and voids. Electromigration is driven by the force of the electron wind, which involves the exchange of momentum between electrons and metal atoms, propelling the atoms to leave their original location and migrate elsewhere. The dependability of wire bonding is further undermined by the aggregation of atoms at the anode and the presence of voids at the cathode, necessitating a comprehensive inquiry into the reliability of wire bonding with respect to high temperature and electromigration challenges [12].

Significant endeavors have been undertaken to address the aforementioned challenges, with wire alloying and over-pad metallization (OPM) being the most popular approaches. In the former, Gam et al. aged alloyed Au bonding wires—Au-1 wt.% Cu wire and Au-1 wt.% Pd wire—which were bonded on Al pads. It turns out that Cu and Pd additions to the Au bonding wire slowed down interfacial reactions and crack extensions due to the formation of a Cu-rich layer and a Pd-rich layer at the interface [13]. In a comparative study, Liu et al. established that the 0.8 mils 80% Au, 15% Ag, and 5% Pd (wt.%) alloyed wire had one order of magnitude lower intermetallic compound growth rate than 99.9% Au wire and displayed superior bonding reliability [14]. With regard to OPM, Ng et al. aimed to investigate the impact of utilizing Electroless Nickel/Electroless Palladium/Immersion Gold (Ni/Pd/Au) and Electroless Nickel/Immersion Gold (Ni/Au) plating on Au wire bonding reliability. The researchers discovered that the wire bonding parameters, wire pull, and ball shear strength of the two plating techniques were similar. Excellent ball shear strength in 8 gF/mil2 was attained, which was 25% greater than bonding on the Al pad [15]. Additionally, Wu et al. analyzed the reliability of thin Cr/Au metal coatings with thicknesses of 50/300 nm and 50/400 nm and reported that thicker coatings displayed superior high-temperature resistance [16].

Despite significant efforts to investigate the reliability of OPM bonding at high temperatures, there remains a knowledge gap regarding the application of OPM structures in the field of electromigration. Therefore, the principal aim of this project is to conduct experiments and simulations to examine the high-temperature electromigration reliability of Au-Al bonding structures and their optimized OPM structures, with the intention of establishing a theoretical foundation for the promotion of OPM structures in high-temperature fields. Traditionally, high-temperature aging is employed in the reliability investigations of Au-Al bonding, followed by the shear stress test, tensile test, and microscopic characterization by Energy Dispersive X-ray Spectroscopy (EDX) and Scanning Electron Microscope (SEM), but these have limitations in examining the various stages of the aging process. Moreover, current aging tests employing resistance measurements with ball on ball contact may not accurately reflect the reliability of wire bonding under electromigration conditions, as reliability of this model cannot be guaranteed [17]. To address these issues, a novel structure utilizing the Kelvin Four-terminal sensing resistance approach has been designed to enable precise measurement in this study.

In addition, the driving mechanism of electromigration can be better understood through Finite element analysis (FEA), which exists two main analysis tools: Atomic Flux Divergence (AFD) and Atomic Density Integration (ADI). The ADI approach is based on the law of vacancy conservation and can predict the location of electromigration damage in homogeneous metals by considering the effects of the electron wind force, temperature gradient, stress gradient, and vacancy concentration gradient [18,19]. However, its application to the analysis of heterogeneous systems is exceedingly difficult. In contrast, the AFD can provide a relatively simple way to predict the damage location by calculating the atomic flux dispersion under each driving force while ignoring the effect of vacancy concentration [20,21]. It has been shown that the atomic fluxes induced by vacancy concentration are smaller than those induced by stress, temperature specificity, and other factors [22]. Therefore, the AFD method is more suitable for analyzing the overall structure of heterogeneous systems.

The high-temperature electromigration test at 250 °C was executed on both Au-Al bonding and OPM structures, which revealed a dramatic escalation in the relative resistance change in the Au-Al bonding structure after 165 h. In contrast, the NiPdAu and CrAu structures exhibited relative resistance changes of only about 2.98% and 46.7% of that of the Au-Al structure demonstrate that the utilization of homogeneous systems could significantly reinforce the reliability and longevity of bonded structures at high temperatures. SEM and EDX analyses of the aged structures revealed the development of extensive voids and cracks in the Au-Al system, which were aligned with the direction of electron movement. Conversely, the OPM structures emerged only minor voids, and the contact area was nearly completely preserved, without any crack expansion or lateral consumption of Al elements. Additionally, the impacts of current direction and the thickness of NiPdAu on the reliability of electromigration were investigated in this study, which showed that the forward current as well as the addition of the pd layer can enhance the bonding reliability to a certain extent. FEA was conducted on the complete Au-Al bond structure based on the AFD principle, and the results were consistent with both the electron wind theory and the actual situation.

## 2. Materials and Methods

### 2.1. Design of Test Sample and Protocol

Various techniques have been developed for the metal coating of substrates, including sputtering, vapor deposition, electroplating, and electroless plating. To achieve the OPM structures, the vacuum magnetron sputtering process was employed in this study for metal coating. The plasmas generated by an abnormal glow discharge of dilute gas under the action of the electric field bombarded the surface of the cathode target, sputtering out the molecules, atoms, ions, and electrons on the surface of the target. The sputtered particles with certain kinetic energies were shot to the surface of the substrate in a specific direction to form a coating layer on the substrate’s surface.

**Figure 1:**
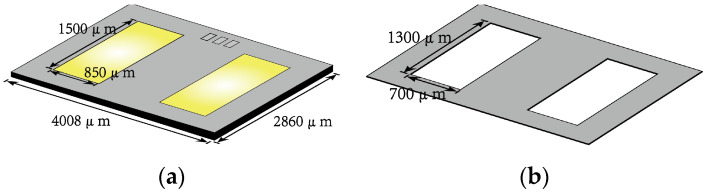

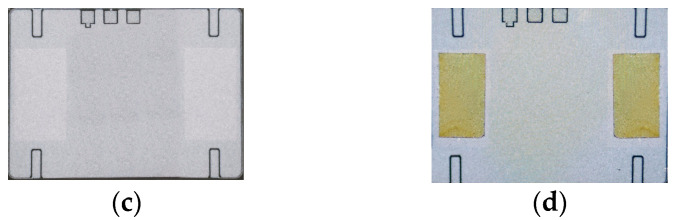
Dimensional parameters of VDMOS device and hard mask. (**a**) Dimensional parameters of VDMOS device; (**b**) Dimensional parameters of the hard mask; (**c**) VDMOS device before sputter coating; (**d**) VDMOS device after sputter coating.

The test samples were prepared with a VDMOS chip, as illustrated in Figure 1a, with dimensions of 4008 μm × 2860 μm and a pad area of 1500 μm × 850 μm. To realize the OPM structure with partially coated metal pads and ensure a certain amount of redundancy, a metal mask measuring 1300 μm × 700 μm and possessing a thickness of 1 μm was employed, as depicted in Figure 1b. Upon completion of the sputtering process, electron microscopy was performed on the samples, revealing that the coating layer was deposited well on the pad area with a substantial amount of redundancy around the pattern. This is demonstrated in Figure 1c,d, which demonstrates that the above technique could provide excellent pattern transfer and satisfy the requirements for OPM structures. During the wire bonding process, careful consideration must be given to the dimensions of the package cavity to prevent excessive wire arc height, which could impact subsequent cap sealing procedures.

**Figure 2:**
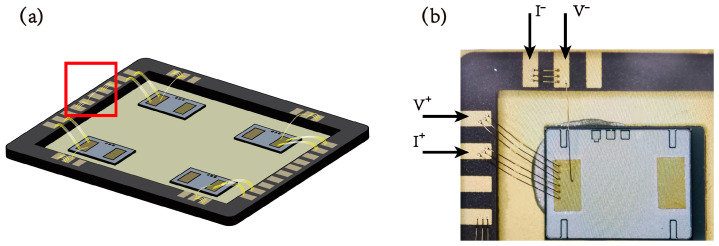
Schematic diagram of wire bonding and testing. (**a**) The layout for wire bonding; (**b**) Design of the test circuits.

The test samples were packaged in Dual In-line package (DIP), with an internal cavity measuring 15.24 μm × 8.99 μm, and four VDMOS chips were included, as illustrated in Figure 2. The bonding parameters employed in this study consisted of an ultrasonic current of 85 mA, a temperature of 160 °C, and a force of 6 g, with a 4 N gold wire having a diameter of 25 μm, which indicated that the OPM bonding process is similar to the conventional Au-Al bonding, and investigations have confirmed that the process window for OPM-treated pads is more expansive [23]. To guarantee the precision of the test results and eliminate resistance fluctuations due to contact between the gold fingers and wires, wire bonding was executed as shown in Figure 2a, with the resistance between the two points monitored and compared as a reference. Furthermore, the test circuit was configured according to Figure 2b, with an applied current of 0.6 A and the test samples exposed to high temperatures of 250 °C in a high-temperature oven. To ensure the dependability of the testing platform, military-grade high temperature wire was employed to lead out the test point for resistance testing outside the oven. Table 1 depicts the sample characteristics and test conditions. A conventional Au-Al bonding configuration served as the reference, while two OPM structures, namely, NiPdAu and CrAu, with thicknesses of 200/100/50 nm and 5/50 nm, respectively, were selected.

### 2.2. Principles of Finite Element Simulation

Electromigration is a diffusion process that occurs when conducting electrons transfer momentum to metal film atoms under high current density-induced stress, leading to the development of open or short circuits. The primary driving forces for atomic mass movement are electron-wind-force-induced migration (EWM), temperature-gradient-induced migration (TM), and stress-gradient-induced migration (SM). The combination of related driving forces eventually results in void formation at various locations [20,24,25,26]. AFD describes the atomic mass transport mechanism and its contribution to electromigration damage, which is calculated for each driving force to determine the site of damage, allowing for an intuitive and convenient analysis of electromigration damage. The relationship between the primary driving forces and AFD is expressed by formulas [27,28]:

(1) $$J_{ewm}=\frac{N}{kT}eZj\rho D_{0}\exp(-\frac{E_{a}}{kT})$$

(2) $$J_{th}=-\frac{NQ}{kT^{2}}D_{0}\exp(-\frac{E_{a}}{kT})\nabla T$$

(3) $$J_{s}=-\frac{N\Omega }{kT}D_{0}\exp(-\frac{E_{a}}{kT})\nabla \sigma$$

(4) $$\nabla \cdot J_{total}=\nabla \cdot J_{ewm}+\nabla \cdot J_{th}+\nabla \cdot J_{s}$$

where $J_{ewm}$, $J_{th}$, and $J_{s}$ are electron wind flux, temperature gradient flux, and stress flux, respectively. $J_{total}$ is the total diffusion flux, N is the atomic concentration, k is the Boltzmann constant, e is the electron charge, T is the thermodynamic temperature, ρ is the temperature-dependent resistivity, j is the current density vector, $D_{0}$ represents the self-diffusion coefficient of atoms in the metal wire, Z is the effective charge number, Q is the heat transfer, Ω is the atomic volume, and σ is the local static stress. The hillocks will develop in the area if the value is negative, but voids or cracks will grow there if the value is positive. The pertinent parameters of the Au elements needed to perform divergence analysis are listed in Table 2.

**Figure 3:**
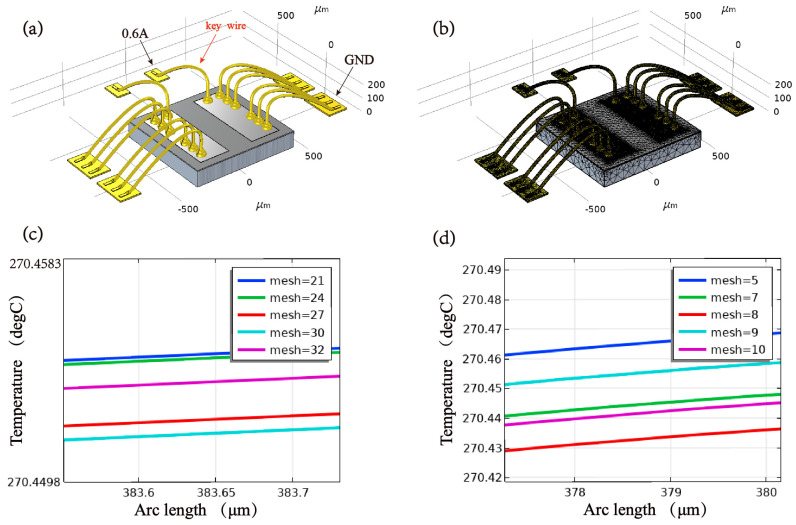
Finite element simulation model and mesh configuration. (**a**) Geometry model; (**b**) Grid partition; (**c**) The impact of the silicon substrate mesh size on the key wire temperature; (**d**) The impact of the wire and pad mesh size on the key wire temperature.

Based on the COMSOL Multiphysics simulation platform, the geometric model and mesh partition of the bonded structure are illustrated in Figure 3. A free tetrahedral mesh was employed to partition the grid, wherein the pad and wire regions were endowed with a minimum element size of 10, a maximum element growth rate of 1.45, and aspect ratios of 0.6 for narrow regions. The mesh partition of the silicon substrate, on the other hand, was implemented, with a minimum element size of 32 and an element growth rate of 1.5, and statistical analysis revealed that the entire mesh comprised 118,835 elements. In the context of electrostatic field boundary conditions, a linear resistivity conduction model was employed to comprehensively account for the temperature effect, as depicted in Figure 3a, in which a current source terminal of 0.6 A and a ground terminal were established. In terms of solid heat transfer boundary conditions, convection heat flux was adopted, with the heat transfer coefficient set at $5\;W/\left(m^{2}\cdot K\right)$ and ambient temperature set at 250 °C. Given that the chip was affixed by silver paste, fixed constraint boundary conditions were designated at the chip’s bottom. In the multi-physical field conditions, electromagnetic heat and thermal expansion were selected to enable the coupling of physical fields. As shown in Figure 3c,d, when the mesh size of the silicon substrate or the pad region is changed, the key wire’s temperature fluctuates only by 0.04 °C or even 0.008 °C. This indicates that the influence on the overall structural analysis was minimal, which proves the rationality of the mesh setting.

In view of the fact that temperature has a significant impact on material resistance, it is necessary to evaluate the characteristics of material resistance with respect to its linear rate of change with temperature. Additionally, in order to consider the effect of temperature on the dimensions and size of the material, it can be quantified by the coefficient of thermal expansion and elastic modulus. Accordingly, to enhance the precision of finite element simulations, it is crucial to adjust the physical parameters of materials, as presented in Table 3.

## 3. Results

### 3.1. Effect of Current Parameters on the Reliability of Au-Al Bonding

**Figure 4:**
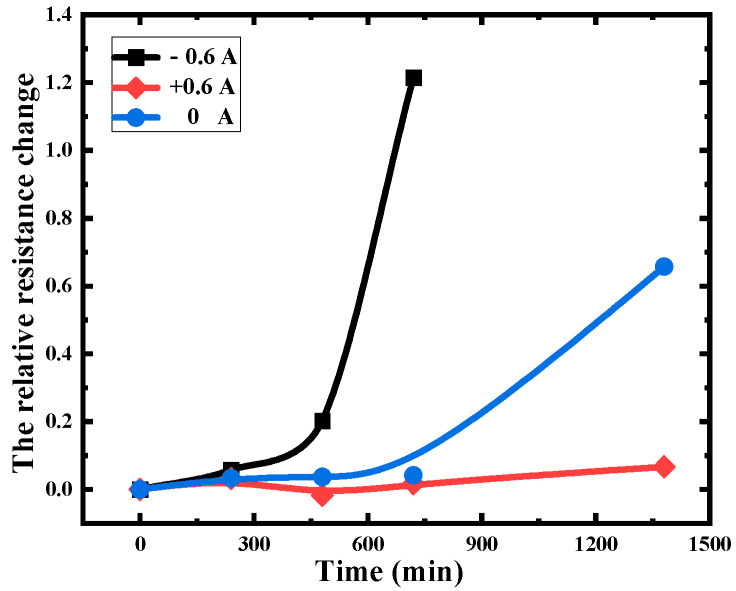
Polarity effect curve of Au-Al bonding (±0.6 A, 0 A).

Electromigration, a prominent failure mechanism in heterogeneous systems, induces the movement of electrons under an electric field, resulting in the formation of IMCs that can have deleterious effects on device functionality. In such systems, the directionality of electromigration is a critical consideration due to the presence of diffusion caused by atomic chemical potentials. The polarity effect of electromigration is that the driving direction of electromigration coincides with atomic diffusion, thereby accelerating the diffusion of atoms and promoting the formation of IMCs, ultimately leading to degradation of the bonding lifetime [32,33]. Conversely, if the electromigration’s driving direction opposes that of metal atoms, which hinders atom diffusion and delays the formation of IMCs, thus enhancing bond strength and the system’s lifetime to some extent. The resistive properties of the interface were investigated as a function of aging time under three different applied currents (+0.6 A, −0.6 A, 0 A), which was defined as the forward current flowing from the gold ball to the Al pad with the results presented in Figure 4. It can be observed that the rate of resistance change under the forward current is significantly lower compared to that under the reverse current stress and the condition of solely high temperature stress. Particularly under the reverse current stress, the relative resistance change rate showed a sharp deterioration.

**Figure 5:**
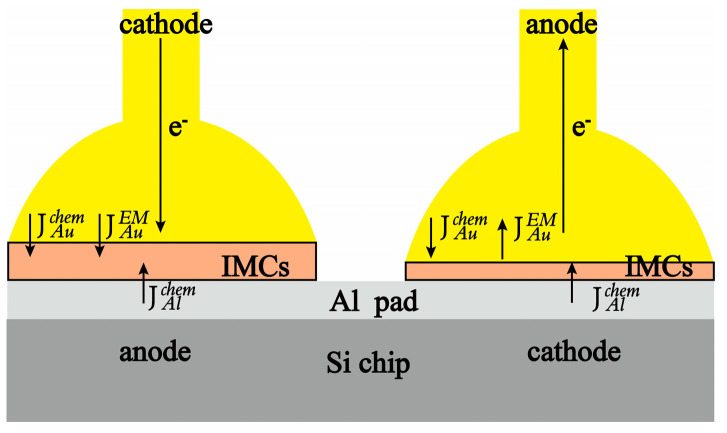
Flux distribution at the Au-Al bonding interface under electromigration.

As shown in Figure 5, the direction of electromigration driving force, represented by $J_{Au}{}^{EM}$, aligns with the direction of electron wind force. $J_{Au}{}^{chem}$ and $J_{Al}{}^{chem}$, on the other hand, represent the atomic diffusion driving forces for Au to Al diffusion and Al to Au diffusion, respectively. In the presence of the electron wind, atom movement towards the anode generates compressive stress, which reduces vacancy concentration on the anode side and increases it on the cathode side, which causes the flow of vacancies from the anode to the cathode, ultimately leading to the aggregation to form voids. 

### 3.2. Electromigration Testing of Au-Al and OPM Structures

**Figure 6:**
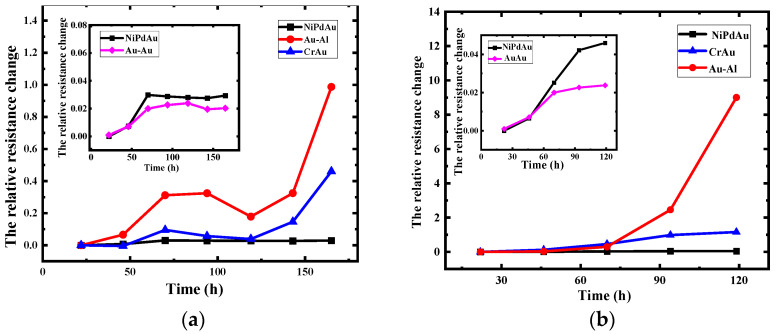
The variation of relative resistance change with aging time. (**a**) 0.6 A, 250 °C; (**b**) 250 °C.

In Figure 6, the influence of temperature and current intensity on the relative resistance change in various bonded structures are investigated. In Figure 6a, the results indicate that the relative resistance change in the Au-Al bonded structure increases significantly to 98.7%, which is 2.14 times and 33.6 times higher than that of the CrAu structure and NiPdAu structure, respectively. This observation may be associated with two distinct failure mechanisms, in which the first stage produces thermal, electrical, and stress-driven intermetallic compounds that alter the structure’s resistance. Subsequently, the expansion of voids, caused by the movement of metal atoms, leads to a significant rise in resistance. Notably, the OPM structure exhibits delayed failure compared to the Au-Al bonding structure, indicating that the presence of the OPM structure impedes the mutual diffusion of metal atoms, delaying void formation and enhancing reliability. In addition, the interfacial resistance between the gold fingers and gold wires only varied by 2% near the end of the aging process, stabilizing as the process progresses. This phenomenon is viewed as a minor imperfection arising from the bonding process or measurement errors, and its effect on the analysis may be ignored. A comprehensive analysis suggests that the OPM structure, formed by the contact of homogeneous metals, can inhibit the diffusion of metal atoms in the initial aging stages, which can delay the accumulation of voids and the propagation of cracks in the later stages.

Aside from investigating electromigration, this study also delves into the effects of high-temperature stress on the relative resistance change in various structures, which reveal that OPM structures remain highly reliable even under such harsh conditions, as shown in Figure 6b. After an exposure period of 70 h, the relative resistance growth rate of the Au-Al bonding structure reaches 29.78%, leading to a rapid increase in interface resistance. However, when compared to the results obtained under forward current, both the Au-Al bonding structure and OPM structure exhibited significant degradations of interface resistance, consistent with the polarity effect principle of electromigration. It is worth noting that the data do not exhibit a “double-slope” distribution pattern at this stage, due to the rapid rate of interface degradation under high-temperature stress.

**Figure 7:**
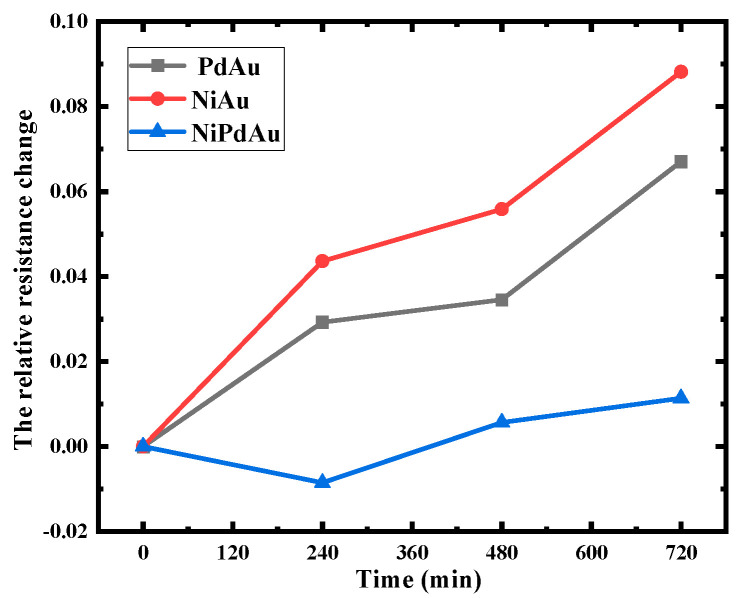
Relative resistance change rates of NiPdAu OPM structures with different thicknesses.

To gain a comprehensive understanding of factors influencing the electromigration resistance of NiPdAu OPM structures, high-temperature storage tests were conducted on structures with varying thicknesses of 200/100/50 nm, 200/0/50 nm, and 0/100/50 nm were selected, with results shown in Figure 7. Remarkably, the NiPdAu structures with thicknesses of 200/0/50 nm and 0/100/50 nm exhibited relative resistance growth rates of 8.82% and 6.70%, respectively, upon completion of the experiment. In contrast, the structure with a thickness of 200/100/50 nm demonstrated a significantly lower resistance growth rate of only 1.11%, which implies that the presence of the Pd layer improved the reliability of the structure. This phenomenon arises from the mutual diffusion of atoms, which becomes even more complex when the current traverses the material contact surface, leading to the formation of brittle compounds [34]. Furthermore, the migration of Ni atoms to the Au layer is known to undergo an oxidation reaction that leads to the formation of “black nickel” and consequent degradation of the bonding ball, which is significantly mitigated by the presence of the Pd layer as the formation of a stable alloy layer with the Au layer [35].

### 3.3. Microscopic Characterization

**Figure 8:**
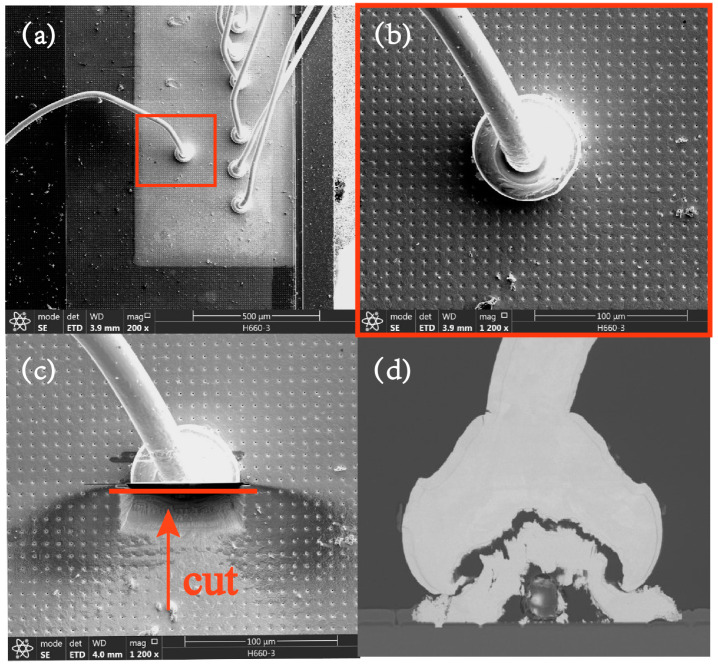
Schematic diagram of EDX and SEM sample preparation. (**a**) Selected key bonding structure; (**b**) Image of enlarged bond structure; (**c**) Image of cutting position; (**d**) Image of bonding interface morphology.

To further assess the reliability of the bonding structure, SEM and EDX analyses were employed to analyze the appearance and chemical composition of the Au-Al bonding interface post testing (0.6 A, 250 °C). Prior to microscopic examination, the sample was prepared via cutting. The Focused Ion Beam (FIB) technique was selected in this investigation owing to the possibility of generating brittle compounds and cracks in the samples during a prolonged aging test, thereby mitigating harm in the sample preparation process. To guarantee the precision and comprehensiveness of the analytical samples, the analysis interface was identified at the central location of the bonding, as illustrated in Figure 8.

**Figure 9:**
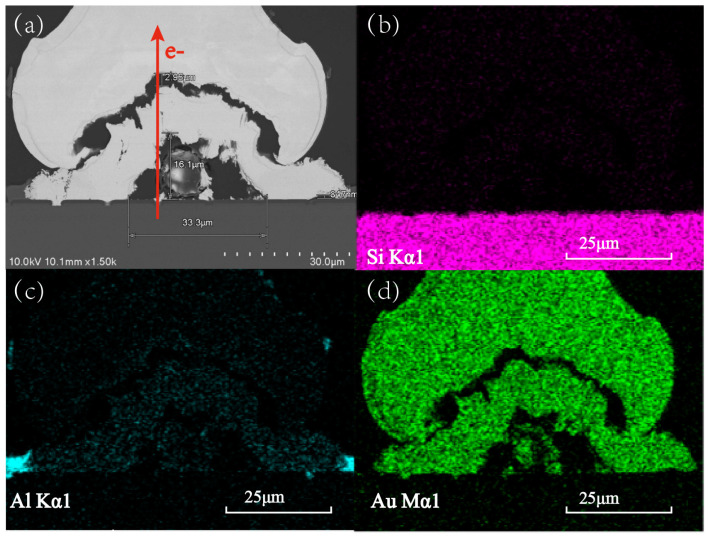
The SEM and EDX images of the annealed Au-Al bonded structure. (**a**) SEM image of the Au-Al bonded interface; (**b**) EDX image of Si element in bonded interface; (**c**) EDX image of Al element in bonded interface; (**d**) EDX image of Au element in bonded interface.

The SEM and EDX results of the Au-Al bonding structure, presented in the accompanying Figure 9, which highlights the presence of notable voids in the bonding interface, measuring approximately 16.1 μm, expanding into more extensive areas of continuous cracks. This occurrence constitutes the principal cause of a sharp rise in the relative resistance change observed at the later annealing stage. Based on the EDX analysis, the Al components in the pad region have been completely depleted, with even Al atoms beyond the pad region being consumed, meaning the significant degradation of the bonding structure’s reliability. Moreover, the crack expands in the same direction as electron motion is attributed to the application of high current intensity during the aging process, consistent with the electron wind theory. When a high-intensity current is applied, electrons gain energy from the electric field and exchange momentum with metal atoms. The atoms that gain energy will break out from their lattice and create voids, which grow over time and produce fractures, thereby reducing the bonding surface at the Au-Al contact and the bonding strength.

**Figure 10:**
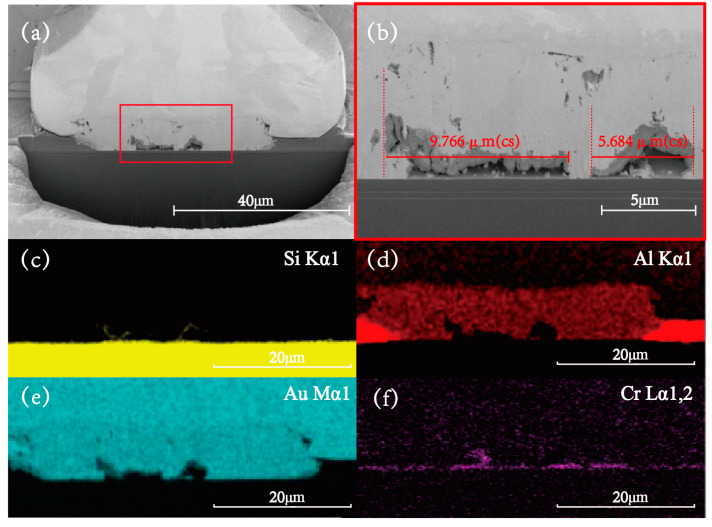
The SEM and EDX images of the annealed CrAu OPM structure. (**a**) SEM image of the CrAu OPM bonded interface; (**b**) SEM image of the local void shape; (**c**–**f**) EDX scan image of the bonded interface.

The microscopic morphology of the CrAu structure is illustrated in Figure 10, which presents a conspicuous IMCs at the bonding interface, indicating interdiffusion between the atoms, which is supported by the findings in Figure 10d,e. Additionally, voids measuring 9.766 μm and 5.648 μm were observed at the interface, which explains the rise of the second period of interfacial resistance. In contrast to the Au-Al bonding system, the CrAu OPM structure does not exhibit significant lateral consumption and continuous cavities, implying that the CrAu structure is more resistant to electromigration under similar test conditions. However, the EDX images showed that the Cr element also diffused during long-term aging, mainly due to the thinness of the Cr layer, leading to limited resistance to electromigration. Thus, for practical engineering applications, the OPM process should be optimized with actual requirements in mind.

**Figure 11:**
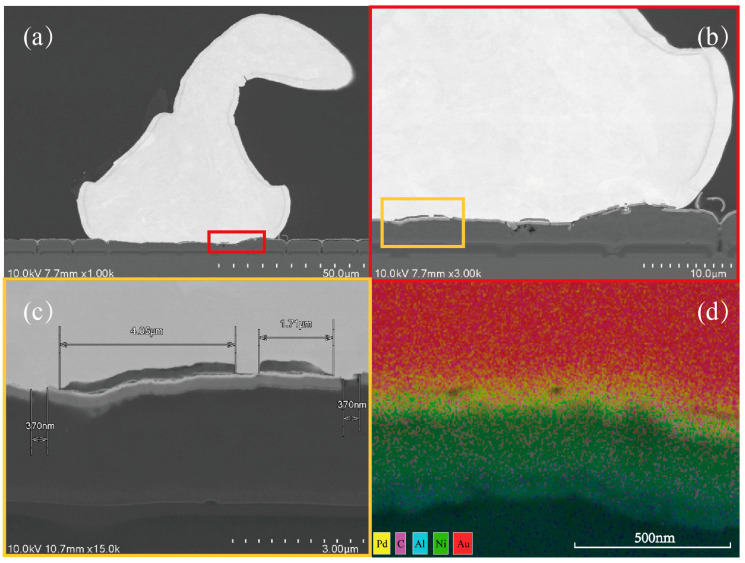
The SEM and EDX scans of the annealed NiPdAu bonded structure. (**a**) SEM picture of the overall bonded structure; (**b**,**c**) SEM image of the local void shape; (**d**) EDX scan image of the bonded interface.

Although NiPd is known to enhance bond strength, it is associated with the formation of “black pads”, which are nickel surfaces that have been passivated or corroded, leading to brittle failure at the bonding contact. To address this issue, a palladium layer is applied to the underlying nickel layer before surface gold plating for NiPdAu finishes. The palladium deposit is characterized by its smooth, amorphous, and homogeneous nature, forming a dense barrier layer that effectively impedes the diffusion of underlying nickel to the gold. Although the palladium deposit in the OPM is relatively thin, measuring approximately 0.06 μm, previous research has shown no substantial degradation of the bonded structure following surface treatment, which is consistent with test results in this study [36]. Figure 11 presents SEM and EDX scan images of the NiPdAu structure, demonstrating that the bonded structure maintains a large area of contact between the gold ball and the pad. The EDX scans further confirmed that the transition lines between the layers were visible, indicating that although some diffusion may have occurred at the intersection of the materials, a dense barrier layer was generated, effectively inhibiting further metal atom diffusion and extending the service life of the NiPdAu structure.

### 3.4. Finite Element Analysis

**Figure 12:**
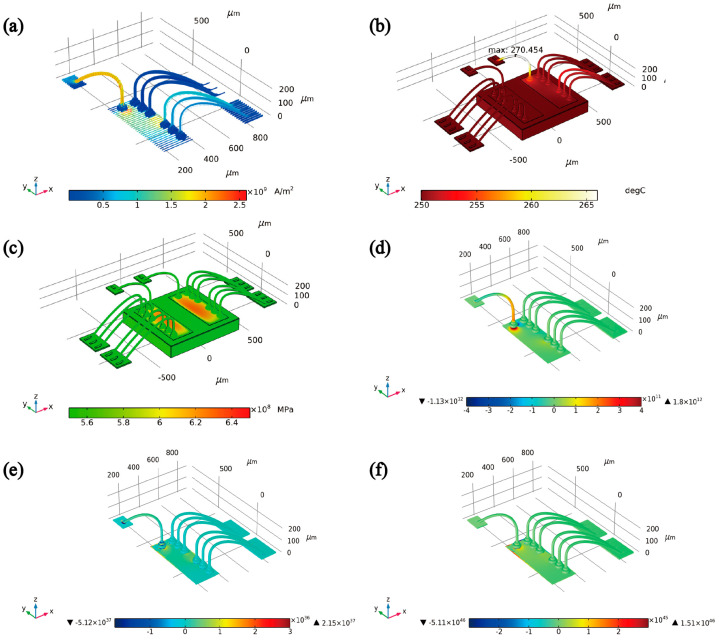
The Distribution of physical fields and the AFD fields. (**a**) The distribution of current density; (**b**) The distribution of temperature; (**c**) The distribution of stress; (**d**) The distribution of electromigration AFD; (**e**) The distribution of thermal-migration AFD; (**f**) The distribution of stress-migration AFD.

Figure 12 depicts the basic physical field distribution and the AFD field distribution of the Au-Al bonding structure. Specifically, Figure 12a illustrates the current density distribution diagram of the structure, wherein the path of electrons from the anode to the cathode is shown to be non-uniform, with electrons congregating at the Au-Al bonding interface and the wire’s neck due to the complex structure’s size and shape, resulting in an amplified current intensity. It can be inferred that the bonding interface is the most vulnerable area for electromigration damage when only considering the electron wind force. Additionally, Figure 12b displays the temperature distribution diagram of the structure, revealing that the key wire’s temperature increased by 20 °C compared to the ambient temperature due to the elevated current intensity, thereby implying that the packaging system is subjected to higher temperature stresses than the environment during operation. The stress distribution diagram of the structure, shown in Figure 12c, indicates that stress is primarily concentrated in the pad area, as a result of the mismatch in lattice constants and the difference in thermal expansion coefficient of materials.

Figure 12d displays the atomic flux divergence distribution caused by the electron wind force, which depicts a negative AFD at the input end indicating accumulation and a positive AFD at the output end signifying divergence to form a hole, in line with the electron wind theory. The thermal migration flux divergence distribution, shown in Figure 12e, highlights that the damage from thermal migration is concentrated mainly at the bonding interface and bonding ball, with AFD present at the junction of the gold finger and wire due to the temperature gradient. Moreover, the stress migration AFD, depicted in Figure 12f, caused by the stress gradient reveals that the damage location is mainly focused at the bonding interface, and there is no AFD at the junction of the gold finger and wire due to minimal contact differences between homogeneous metals according with the actual situation.

In light of the comprehensive analysis, the interface of Au-Al bonding emerges as the most vulnerable and focal point of electromigration damage, whereas the neck of wire and gold finger also demonstrate the presence of electromigration damage in line with the actual situation. Furthermore, the results reveal that the distribution of atomic flux divergence, caused by various driving forces, follows the order of $\nabla \cdot J_{ewm}<\nabla \cdot J_{th}<\nabla \cdot J_{s}$, which underscores that the electromigration force propels the mass migration of metal atoms, engendering the generation of Joule heat and voids, with subsequent temperature and stress gradients in metal lines. Insofar as the gradient functions as the impetus for the driving force, its existence triggers the mass transport of metal atoms, culminating in the accumulation and expansion of voids through the combined action of thermal migration and stress migration, thereby compromising the reliability of the bonded structure as a consequence.

## 4. Conclusions

This study proved that the OPM structure, the contact between homogenous metals, offers more reliable resistance to electromigration in conditions of elevated temperature. This presents a significant advantage in the field of device packaging, as the traditional wire bonding process can be employed in OPM structures, thereby extending the application of wire bonding to high-temperature scenarios within a defined process window.

The results obtained from the high-temperature annealing tests indicated a two-step increase in the interfacial resistance of the samples, which was primarily attributed to the existence of two distinct failure modes. By considering the same failure criteria, the lifetimes of CrAu and NiPdAu were increased by 2.14 and 33.6 times, respectively, compared to conventional bonded structures. The SEM and EDX results revealed the existence of cracks at the Au-Al bonding interface with an extension direction that is consistent with the direction of electron motion, leading to a significant rise in the interfacial resistance at later stages. Conversely, the bonding interface of the OPM structure retained a better contact state. The movement of metal atoms in the active state was propelled by electron wind forces under high temperature and high current, resulting in the formation of significant voids and cracks, which was the fundamental failure mechanism of the bonded interface. However, the OPM structure is observed to delay this process, thus increasing the high-temperature lifetime of the bonded structures. In addition, simulation results of the complete Au-Al structure indicated that the electromigration damage mainly concentrated at the interface of bonding, the junction of the finger and wire, and the neck of the wire, which was consistent with the electron wind theory and practical situations. The findings of this research, particularly in the context of high-temperature sensors, have significant reference implications for addressing the challenges associated with Au-Al bonding to enhance the packaging reliability of these devices.

## Figures and Tables

**Table 1:** The test conditions and sample characteristics.

Materials	Sample	Thickness	Temperature	Current	Time
Al	S1	−	250 °C	0.6 A	165 h
NiPdAu	S2	200/100/50 nm	250 °C	0.6 A	165 h
CrAu	S3	5/50 nm	250 °C	0.6 A	165 h

**Table 2:** The relevant parameters of the material.

Parameter	Unit	Value	
		Au	Al [29]
$\;E_{a}$	eV	0.785	0.87
Z	−	3.7	−4
$D_{0}$		$1.075\times 10^{-5}$ [30]	$5\times 10^{-8}$
Q	kJ/mole	176.9 [30]	−0.0867
Ω	$m^{2}$	$7.036\times 10^{-30}$	$1.658\times 10^{-29}$

**Table 3:** The critical parameters of the material.

Material	Elastic Modulus (GPa) [31]	Poisson’s Ration	Thermal Conductivity (W·m^−1^·K^−1^)	Resistivity (10^−8^·Ω·m)	Thermal Expansion (K^−1^) [29]					
					200 K	300 K	400 K	500 K	600 K	700 K
Al	75.34−0.034T	0.35	240.0	3.24×(1+3.5× $10^{-3}\left(T-303\right))$	20.3	23.23	25.20	26.4	28.4	30.90
Au	82.62−0.03T	0.44	317	2.2	$4.2\times 10^{-5}$	$4.4\times 10^{-5}$	$4.7\times 10^{-5}$	$4.9\times 10^{-5}$	$5.3\times 10^{-5}$	$5.9\times 10^{-5}$
Si	71	0.16	1.75	4.4	2.24	2.64	3.20	3.50	3.70	3.90

## Data Availability

The data presented in this study are available on request from the corresponding author. The data are not publicly available due to privacy.
